# Supplementary material for: Causal Relationship between Aging and Anorexia Nervosa: A White-Matter-Microstructure-Mediated Mendelian Randomization Analysis
Source: Biomedicines. 2024 Aug 16;12(8):1874. doi: 10.3390/biomedicines12081874 (PMC11351342; doi:10.3390/biomedicines12081874)
Supplement: Supplementary file 1 [file biomedicines-12-01874-s001.zip › Supplementary Figure/Figure S8/Leaveoneout plot of MR analysis for TL to ubm-b-1469.pdf]

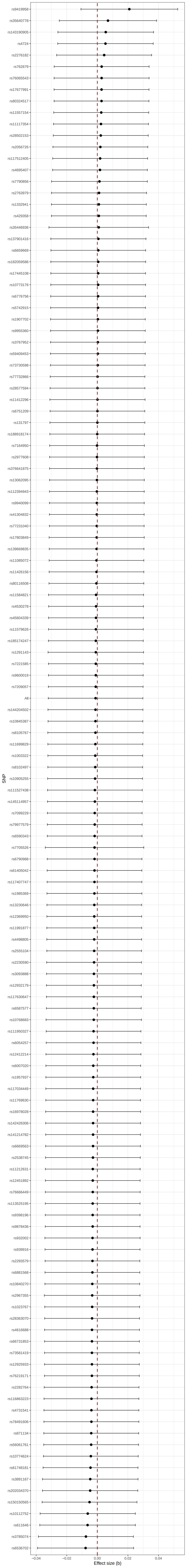

rs9419958
rs35640778
rs143190905
rs4724
rs2276182
rs762679
rs76065543
rs17677991
rs80324517
rs11557154
rs11117354
rs28502153
rs2056726
rs117512405
rs4695407
rs7790856
rs2763979
rs1332941
rs429358
rs35446936
rs137901416
rs6659669
rs182059586
rs17445108
rs10773176
rs6776756
rs5742915
rs1907702
rs9955360
rs3767952
rs59409453
rs73730598
rs77732866
rs28577594
rs11412296
rs6751209
rs131797
rs188918174
rs7164950
rs2977608
rs376641875
rs13062095
rs112394943
rs9940099
rs41304832
rs77231040
rs17803849
rs139669835
rs11085072
rs11426156
rs80116508
rs11584821
rs4530278
rs45604339
rs11579626
rs185174247
rs1291143
rs7221585
rs9600019
rs7209057
All
rs144204502
rs10845387
rs8105767
rs11699829
rs1003322
rs8102497
rs109052555
rs111527438
rs145114957
rs7099229
rs79977579
rs6590343
rs7705526
rs6790988
rs61405042
rs117407747
rs1985369
rs13230646
rs12369950
rs11991877
rs4498805
rs2555104
rs2230590
rs3093888
rs12932179
rs117630647
rs6587577
rs10768683
rs111950327
rs6054257
rs12412214
rs6007020
rs1957937
rs117034449
rs11769630
rs16978028
rs142426306
rs141214782
rs6669563
rs2538745
rs11212631
rs12451892
rs76666449
rs113525195
rs9398196
rs9878436
rs932002
rs939916
rs2293579
rs6881568
rs10840270
rs2967355
rs1023767
rs28363070
rs4616688
rs66731853
rs73581419
rs12925933
rs76219171
rs2282764
rs116863223
rs4731541
rs78491606
rs871134
rs56061761
rs10774624
rs61748181
rs3891167
rs202034370
rs150150565
rs10112752
rs611646
rs3785074
rs6536702
SNP
-0.04
-0.02
0.00
0.02
0.04
Effect size (b)
